# The future of medicine in a One Health world

**DOI:** 10.1371/journal.pmed.1005042

**Published:** 2026-03-27

**Authors:** John L. Gittleman

**Affiliations:** 1 Nicholas School of the Environment, Duke University, Durham, North Carolina, United States of America; 2 Odum School of Ecology, University of Georgia, Athens, Georgia, United States of America

## Abstract

Long recognized as a breakthrough synthetic approach, One Health has been slow and piecemeal to infiltrate medical fields. In this Perspective, John Gittleman argues that it’s time for change by taking on a new patient - the environment.

## Teaser

Long recognized as a breakthrough approach, One Health has been slow and piecemeal to infiltrate medical fields. Growing evidence suggests that it’s time for change by taking on a new patient—the environment.

The One Health approach, recognizing animal–human–environmental interconnections and their implications for planetary and human health, has been percolating for decades, but now by necessity it’s being taken seriously. Environmental disasters are rampant and increasing: Between 1980 and 2024, the US suffered 403 billion-dollar weather and climate disasters, costing more than $2.9 trillion in direct costs and claiming nearly 17,000 lives [1]. Between 2001 and 2018, residents of 2,711 counties, covering over 75% of the US population, experienced at least one large flood during the study period [2] due to heavy rain, snowmelt, or tropical cyclones. These floods were associated with increases in all-cause mortality, including a 15.3% increase in injury death rate, as well as increases in deaths attributable to infectious disease (3.2%) and cardiovascular disease (2.1%).

**Fig 1:**
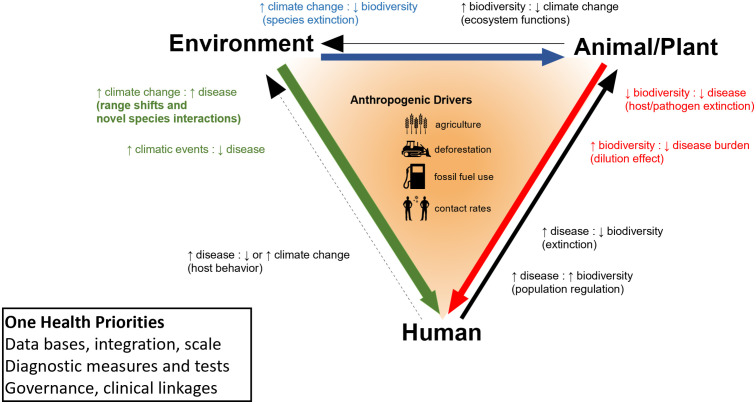
Interconnections between One Health and medicine. A conceptual model of One Health interconnections of animals/plants, humans, and the environment as influenced by anthropogenic factors such as transportation, climate, and habitat structures which effect levels of interconnected contact. Hypothetical examples are shown (outside of the triad) for One Health impacts via increases and decreases of various elements. Medical advances employing One Health approaches will require improvements in database availability, diagnostic procedures, and governance (see text).

Increasingly, environmental disasters involve “knock-on” interconnections of animal, human, and environmental factors (Fig 1) [3]. These linkages aren’t new, but the opportunities resultant from squeezing of space and human density are: Pure undisturbed planetary habitat is estimated to be as low as 2.8% of the world’s land surface [4], about equivalent to the collective size of Europe. Habitat loss and degradation increase the rate of animal–human contacts and synergies between intense environmental change and growing human populations [5]. The approach that recognizes the inevitable effects of this compression is One Health.

A recent publication, *The Lancet’s* One Health Commission Report [6], now confronts these problems with a broad road map to integrate environmental and socioecological factors into medicine. The document describes historical developments and usages of the One Health concept, strengths (epidemiology, infectious diseases) and deficiencies (non-communicable diseases, plants), database needs and surveillance methods, and governance and health systems. Importantly, the report concludes with 10 recommendations ranging from One Health governance to integrated surveillance systems for improving sustainable food and health systems. While an ambitious undertaking due to many unyielding constraints (e.g., medical silos, insurance systems, costs, etc.), this can be made possible with bold and collaborative approaches across all sectors. In light of this, below I outline recommendations for how a One Health approach can be facilitated, including through the engagement of “One Health Practitioners” (OHPs).

## One Health Practitioners

Analogous to doctors, physicians, and other healthcare workers specializing in a given field such as surgery, psychiatry, infectious diseases, etc., I propose that an equivalent role for One Health approaches is imperative, in the form of OHPs. While public health professionals have collaborated with medical environmental scientists and ecologists to address environmental and socio-ecological determinants of health [7], such data needs better integration into healthcare settings to more effectively evaluate medical problems and inform treatment procedures and policies related to environmental issues impacting health.

In this respect, OHPs with environmental scientific expertise directly linked to human health would work with physicians in areas that are increasingly faced with environmental casualties. Providers could operate within a team to bring specific information and data relevant to a particular problem to anticipate and respond to medical emergencies resultant from environmental causes such as floods, wildfires, heatwaves, etc. There is also a strong case to be made for healthcare professionals to receive “ecomedical” training in One Health as an integral part of curricula to build awareness and improve skills of health professionals [8,9]. It will be critical to gradually assimilate OHPs into teams wherein communication is clear, effective, and specific to the One Health problems in a given community. Below, I outline three example environmental subfields in which OHPs could be integrated to facilitate One Health approaches that inform medical practice.

## Integrating environmental subfields into medicine with One Health Practitioners

The first example of an environmental subfield with direct health implications is fire ecology. Using tools such as remote sensing, fire ecology specialists can detect and predict burned area severity and fire risk of an area, particularly those traversing complex habitats (whether natural or constructed). With wind velocity, land cover (vegetation index), and ground temperature data, machine learning models can predict country wildfire spreading from 1 to 5 days [10]. Working with OHPs, this information could be used to inform local hospitals and healthcare facilities to help them prepare for and treat conditions that may be exacerbated by wildfire smoke (such as asthma, chronic obstructive pulmonary disease, and respiratory infections), as well as inform anticipatory public health responses to mitigate and minimize the negative health effects of wildfire smoke.

Another obvious field is disease ecology [11], which rapidly emerged during the COVID-19 pandemic. Here, the origins, spread, and containment of diseases like West Nile, Lyme disease, and malaria are directly linked to environmental factors involving habitat fragmentation, climate change, and invasive species, combined with anthropogenic factors of population growth, urbanization, and world travel and trade. Armed with comprehensive databases (e.g., GIDEON [12]), disease ecology is an excellent role model for successfully bringing together interdisciplinary research from different fields (ecology, evolution, epidemiology, applied statistics) and approaches to better understand One Health and address the impact of human-driven environmental change on diseases. Indeed, the benefit-cost of predicting viral diseases reaches staggering economic savings, in the billions of dollars, not to mention the lives saved [13]. Here, OHPs will bring specific advice on how and under what environmental exposures (e.g., temperature, humidity, PM2.5, PM10) infectious diseases or noncontagious diseases such as skin or respiratory conditions may occur [14].

Lastly (but not least), is ecotoxicology, a relatively modern discipline, formalized in the 1970s to study multiple effects of toxic elements from toxic inorganic compounds (such as hydrogen cyanide, carbon monoxide, and asbestos), organic compounds (such as carcinogenic benzene), and radionuclides generated from nuclear power plants. Effects from exposure to toxins are complicated, yet we know that fires, floods, and other physically obstructive events release toxins, exposing the liver, kidneys, and potentially other organ systems to high risks of toxin-induced damage. To assist with mitigation strategies and minimizing negative impacts on health, maps of potential toxic substances should be drawn of communities and cities ready to implement before and after environmental disasters, with OHPs (in collaboration with ecotoxicologists) armed to anticipate, investigate and assess the potential impact of toxic substances, such as pesticides, heavy metals, and industrial chemicals on human health and the disrupted environment.

## Prioritizing next steps

Supporting and equipping physicians with well-trained OHPs will improve our ability to adapt to and manage our changing environmental world and its impacts on health. *The Lancet* Report [6] redresses an important aspect of training by pointing out imbalances amongst current One Health animal–human–environmental approaches. For example, in addition to attention on infectious diseases, greater emphasis is needed on issues such as AMR, non-communicable diseases (e.g., cardiovascular, respiratory, neurological health), plant biodiversity, and other currently unknown interconnections (see Fig 1) [3,7,14].

This will not be easy or quickly “fixable”. However, immediate priorities should include:

1. Assembling comprehensive and integrated databases with comparable scaling units to isolate and track interactive connections (e.g., how do increases in temperature differentially impact cardiac or respiratory conditions in animals and humans (see additional examples in [6,14]).
2. Diagnostic tests designed to identify toxins in blood or urine (e.g., mycotoxin tests). Many existing medical tests could be co-opted for detecting infections, assessing organ functions, diagnosing diseases such as cancer or diabetes, guiding treatment options, or monitoring the effectiveness of ongoing care. For example, hypothesized linkages between gastrointestinal health (e.g., gastroesophageal reflux disease, gastric cancer, esophageal disorders, allergies) and soil toxicity, air pollution, or temperature/humidity extremes.
3. Models to predict the likelihood of different kinds of injuries or diseases anticipated from hurricanes or floods, which will be critical for helping physicians respond to One Health problems and, in so doing, employ procedures for suspected disease, abnormalities, or monitoring of treatments.
4. Governance structures spanning local and global scales, as well as political and educational agendas, prioritizing accurate surveillance systems that can inform medical action.

Together, such collaborative approaches and the engagement of OHPs would be a productive step towards addressing One Health problems more effectively. While causes and treatments are being addressed for many debilitating, profound medical problems such as dementia and inflammation, it’s time to include in our preventive arsenals ways to adapt and sustain life within a changing environmental world. One Health must be considered an integral component of modern medicine.

## Abbreviation:

OHPs: One Health Practitioners
